# 5-Bromo-*N* ^3^-phenyl­pyrazine-2,3-diamine

**DOI:** 10.1107/S1600536809028554

**Published:** 2009-07-25

**Authors:** Xiaohui Zhu

**Affiliations:** a School of Chemistry and Chemical Engineering, Taishan Medical University, Tai’an 271016, People’s Republic of China

## Abstract

In the title compound, C_10_H_9_BrN_4_, the dihedral angle between the benzene and pyrazine rings is 61.34 (5)°. Inter­molecular N—H⋯N hydrogen bonds and N—H⋯π inter­actions assemble the mol­ecules into a three-dimensional network structure.

## Related literature

For Cu or Pd catalysed C–N cross-coupling reactions, see: Fors *et al.* (2009 ▶); Liu *et al.* (2007 ▶).
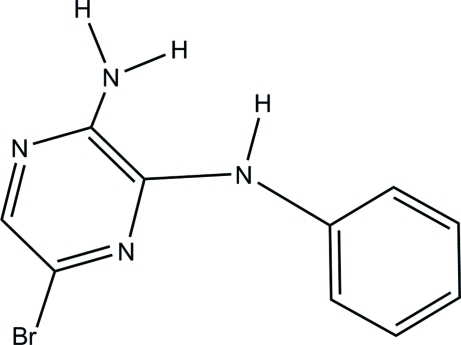


## Experimental

### 

#### Crystal data

C_10_H_9_BrN_4_
*M* *_r_* = 265.12
Monoclinic, 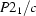

*a* = 7.4834 (8) Å
*b* = 15.4038 (17) Å
*c* = 9.2079 (10) Å
β = 91.307 (2)°
*V* = 1061.1 (2) Å^3^
*Z* = 4
Mo *K*α radiation
μ = 3.85 mm^−1^
*T* = 293 K
0.15 × 0.12 × 0.10 mm

#### Data collection

Bruker SMART APEX diffractometer
Absorption correction: multi-scan (*SADABS*; Bruker, 2005 ▶) *T*_min_ = 0.596, *T*_max_ = 0.700
5494 measured reflections
1871 independent reflections
1555 reflections with *I* > 2σ(*I*)
*R*_int_ = 0.022

#### Refinement

*R*[*F* ^2^ > 2σ(*F* ^2^)] = 0.025
*wR*(*F* ^2^) = 0.059
*S* = 1.00
1871 reflections
140 parameters
H-atom parameters constrained
Δρ_max_ = 0.33 e Å^−3^
Δρ_min_ = −0.33 e Å^−3^

### 

Data collection: *SMART* (Bruker, 2005 ▶); cell refinement: *SAINT* (Bruker, 2005 ▶); data reduction: *SAINT*; program(s) used to solve structure: *SHELXS97* (Sheldrick, 2008 ▶); program(s) used to refine structure: *SHELXL97* (Sheldrick, 2008 ▶); molecular graphics: *SHELXTL* (Sheldrick, 2008 ▶); software used to prepare material for publication: *SHELXL97*.

## Supplementary Material

Crystal structure: contains datablocks I, global. DOI: 10.1107/S1600536809028554/gk2218sup1.cif

Structure factors: contains datablocks I. DOI: 10.1107/S1600536809028554/gk2218Isup2.hkl

Additional supplementary materials:  crystallographic information; 3D view; checkCIF report

## Figures and Tables

**Table 1:** Hydrogen-bond geometry (Å, °)

*D*—H⋯*A*	*D*—H	H⋯*A*	*D*⋯*A*	*D*—H⋯*A*
N1—H1*B*⋯*Cg*1^i^	0.86	2.63	3.436 (3)	157
N1—H1*A*⋯N2^ii^	0.86	2.22	3.084 (3)	169

Symmetry codes: (i) 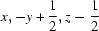; (ii) 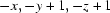. *Cg*1 is the centroid of the C5–C10 ring.

**Fig. 1.**
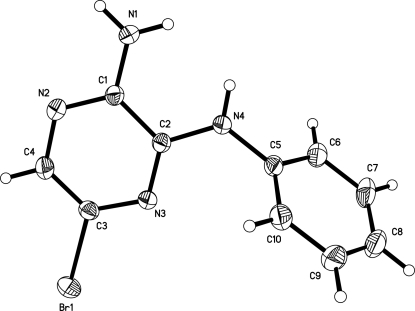
The molecular structure of the title compound, showing 30% probability displacement ellipsoids.

**Fig. 2.**
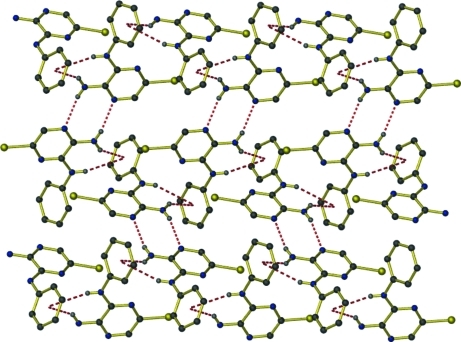
The two-dimensional network structure formed by N—H···N hydrogen bonds and N—H···π interactions.

## supplementary crystallographic information

### Comment

Cu or Pd catalyzed C—N cross-coupling reactions were studied recently (Liu *et al.*, 2007; Fors *et al.*, 2009), which reported that catalysts using certain ligands allow for the C—N cross-coupling reactions. Different to them, we got a cross-coupling product with high selectivity under microwave without catalyst.

Here we report the crystal structure of the title compound. In (I) (Fig.1), the bond length to the bridging NH group are  1.377 (3) and 1.420 (3) Å. Moreover, intermolecular typical N—H···N (N···N 3.084 (3) Å) hydrogen bonds and N—H···π [H···π 2.633 (2) Å]  interaction assemble molecules into a two-dimensional network structure.

### Experimental

5-Bromo-2-aminopyrazine (10 mmol) and aniline (40 mmol) were added to a reaction kettle, then reacted 2 h at 413 K under microwave operation. The product was purified on a SiO_2_ flash column to give a title product (yield 31%). Crystals of the title compound suitable for X-ray analysis were grown from a dichloromethane solution.

### Refinement

The C–H and N–H hydrogen atoms were placed in calculated positions, with distances C—H = 0.93 Å, N—H = 0.87 Å. and *U*_iso_(H) = 1.2 *U*_eq_ (C, N). Initially  positions of the amino H atoms were refined but in the final cycles the N—H distances were constrained to 0.87 Å and all H atoms were treated as riding with *U*_iso_(H) values of 1.5*U*_eq_(C,N).

### Crystal data

**Table:**

C_10_H_9_BrN_4_	*F*(000) = 528
*M**_r_* = 265.12	*D*_x_ = 1.660 Mg m^−^^3^
Monoclinic, *P*2_1_/*c*	Mo *K*α radiation, λ = 0.71073 Å
Hall symbol: -P 2ybc	Cell parameters from 2474 reflections
*a* = 7.4834 (8) Å	θ = 2.6–27.6°
*b* = 15.4038 (17) Å	µ = 3.85 mm^−^^1^
*c* = 9.2079 (10) Å	*T* = 293 K
β = 91.307 (2)°	Block, colorless
*V* = 1061.1 (2) Å^3^	0.15 × 0.12 × 0.10 mm
*Z* = 4	

### Data collection

**Table:**

Bruker SMART APEX diffractometer	1871 independent reflections
Radiation source: fine-focus sealed tube	1555 reflections with *I* > 2σ(*I*)
graphite	*R*_int_ = 0.022
φ and ω scans	θ_max_ = 25.0°, θ_min_ = 2.6°
Absorption correction: multi-scan (*SADABS*; Bruker, 2005)	*h* = −8→8
*T*_min_ = 0.596, *T*_max_ = 0.700	*k* = −17→18
5494 measured reflections	*l* = −10→10

### Refinement

**Table:**

Refinement on *F*^2^	Secondary atom site location: difference Fourier map
Least-squares matrix: full	Hydrogen site location: inferred from neighbouring sites
*R*[*F*^2^ > 2σ(*F*^2^)] = 0.025	H-atom parameters constrained
*wR*(*F*^2^) = 0.059	*w* = 1/[σ^2^(*F*_o_^2^) + (0.025*P*)^2^ + 0.6504*P*] where *P* = (*F*_o_^2^ + 2*F*_c_^2^)/3
*S* = 1.00	(Δ/σ)_max_ = 0.002
1871 reflections	Δρ_max_ = 0.33 e Å^−^^3^
140 parameters	Δρ_min_ = −0.33 e Å^−^^3^
0 restraints	Extinction correction: *SHELXL97* (Sheldrick, 2008), Fc^*^=kFc[1+0.001xFc^2^λ^3^/sin(2θ)]^-1/4^
Primary atom site location: structure-invariant direct methods	Extinction coefficient: 0.0241 (11)

### Special details

**Table:**

Geometry. All e.s.d.'s (except the e.s.d. in the dihedral angle between two l.s. planes) are estimated using the full covariance matrix. The cell e.s.d.'s are taken into account individually in the estimation of e.s.d.'s in distances, angles and torsion angles; correlations between e.s.d.'s in cell parameters are only used when they are defined by crystal symmetry. An approximate (isotropic) treatment of cell e.s.d.'s is used for estimating e.s.d.'s involving l.s. planes.
Refinement. Refinement of *F*^2^ against ALL reflections. The weighted *R*-factor *wR* and goodness of fit *S* are based on *F*^2^, conventional *R*-factors *R* are based on *F*, with *F* set to zero for negative *F*^2^. The threshold expression of *F*^2^ > σ(*F*^2^) is used only for calculating *R*-factors(gt) *etc*. and is not relevant to the choice of reflections for refinement. *R*-factors based on *F*^2^ are statistically about twice as large as those based on *F*, and *R*- factors based on ALL data will be even larger.

### Fractional atomic coordinates and isotropic or equivalent isotropic displacement parameters (Å^2^)

**Table:**

	*x*	*y*	*z*	*U*_iso_*/*U*_eq_	
Br1	−0.05901 (4)	0.351301 (18)	1.09071 (3)	0.05020 (14)	
N1	0.1466 (3)	0.39455 (14)	0.4791 (2)	0.0455 (5)	
H1A	0.1000	0.4388	0.4334	0.046 (8)*	
H1B	0.2400	0.3676	0.4472	0.075 (11)*	
N2	−0.0254 (3)	0.43567 (13)	0.6710 (2)	0.0404 (5)	
N3	0.1484 (2)	0.31467 (12)	0.84959 (19)	0.0319 (4)	
N4	0.3193 (3)	0.27272 (13)	0.6497 (2)	0.0420 (5)	
H4	0.2980	0.2595	0.5591	0.063 (9)*	
C1	0.1017 (3)	0.38587 (14)	0.6195 (2)	0.0317 (5)	
C2	0.1899 (3)	0.32331 (14)	0.7117 (2)	0.0302 (5)	
C3	0.0152 (3)	0.36556 (15)	0.8955 (2)	0.0343 (5)	
C4	−0.0699 (3)	0.42447 (16)	0.8104 (3)	0.0423 (6)	
H4A	−0.1608	0.4579	0.8488	0.051*	
C5	0.4259 (3)	0.21097 (15)	0.7267 (2)	0.0365 (6)	
C6	0.4269 (4)	0.12553 (16)	0.6809 (3)	0.0446 (6)	
H6	0.3565	0.1084	0.6014	0.054*	
C7	0.5329 (4)	0.06590 (19)	0.7538 (3)	0.0564 (8)	
H7	0.5338	0.0084	0.7229	0.068*	
C8	0.6367 (4)	0.0902 (2)	0.8710 (3)	0.0613 (8)	
H8	0.7056	0.0491	0.9209	0.074*	
C9	0.6390 (4)	0.1748 (2)	0.9145 (3)	0.0589 (8)	
H9	0.7108	0.1916	0.9934	0.071*	
C10	0.5348 (3)	0.23602 (18)	0.8419 (3)	0.0468 (6)	
H10	0.5383	0.2938	0.8709	0.056*	

### Atomic displacement parameters (Å^2^)

**Table:**

	*U*^11^	*U*^22^	*U*^33^	*U*^12^	*U*^13^	*U*^23^
Br1	0.0541 (2)	0.0615 (2)	0.03565 (17)	0.00683 (14)	0.01461 (11)	0.00123 (12)
N1	0.0648 (15)	0.0406 (12)	0.0314 (11)	0.0182 (11)	0.0047 (10)	0.0082 (9)
N2	0.0466 (13)	0.0353 (11)	0.0392 (12)	0.0102 (9)	0.0014 (9)	0.0047 (9)
N3	0.0345 (11)	0.0327 (10)	0.0288 (10)	0.0035 (8)	0.0036 (8)	0.0018 (8)
N4	0.0504 (13)	0.0451 (12)	0.0310 (11)	0.0194 (10)	0.0094 (9)	0.0070 (9)
C1	0.0369 (13)	0.0262 (11)	0.0320 (12)	0.0014 (10)	−0.0003 (10)	−0.0008 (9)
C2	0.0330 (13)	0.0265 (11)	0.0312 (12)	0.0008 (9)	0.0013 (10)	0.0001 (9)
C3	0.0375 (13)	0.0359 (13)	0.0297 (12)	−0.0004 (10)	0.0048 (10)	−0.0004 (10)
C4	0.0433 (15)	0.0400 (14)	0.0440 (15)	0.0124 (11)	0.0085 (11)	0.0005 (11)
C5	0.0339 (13)	0.0404 (14)	0.0356 (12)	0.0084 (10)	0.0107 (10)	0.0087 (11)
C6	0.0408 (15)	0.0416 (15)	0.0516 (16)	0.0068 (11)	0.0040 (12)	0.0028 (12)
C7	0.0502 (17)	0.0404 (15)	0.079 (2)	0.0149 (13)	0.0099 (15)	0.0084 (14)
C8	0.0492 (18)	0.069 (2)	0.066 (2)	0.0229 (15)	0.0049 (15)	0.0211 (16)
C9	0.0419 (16)	0.084 (2)	0.0504 (17)	0.0121 (15)	−0.0041 (13)	0.0015 (15)
C10	0.0438 (15)	0.0478 (15)	0.0489 (15)	0.0067 (12)	0.0064 (12)	−0.0016 (13)

### Geometric parameters (Å, °)

**Table:**

Br1—C3	1.906 (2)	C4—H4A	0.9300
N1—C1	1.349 (3)	C5—C10	1.378 (4)
N1—H1A	0.8699	C5—C6	1.382 (3)
N1—H1B	0.8700	C6—C7	1.378 (4)
N2—C1	1.319 (3)	C6—H6	0.9300
N2—C4	1.344 (3)	C7—C8	1.368 (4)
N3—C2	1.320 (3)	C7—H7	0.9300
N3—C3	1.344 (3)	C8—C9	1.364 (4)
N4—C2	1.377 (3)	C8—H8	0.9300
N4—C5	1.420 (3)	C9—C10	1.385 (4)
N4—H4	0.8700	C9—H9	0.9300
C1—C2	1.436 (3)	C10—H10	0.9300
C3—C4	1.349 (3)		
			
C1—N1—H1A	115.8	C3—C4—H4A	119.4
C1—N1—H1B	119.8	C10—C5—C6	119.6 (2)
H1A—N1—H1B	121.8	C10—C5—N4	120.8 (2)
C1—N2—C4	117.7 (2)	C6—C5—N4	119.5 (2)
C2—N3—C3	115.85 (19)	C7—C6—C5	119.5 (3)
C2—N4—C5	124.36 (19)	C7—C6—H6	120.2
C2—N4—H4	114.5	C5—C6—H6	120.2
C5—N4—H4	114.3	C8—C7—C6	120.8 (3)
N2—C1—N1	119.0 (2)	C8—C7—H7	119.6
N2—C1—C2	120.2 (2)	C6—C7—H7	119.6
N1—C1—C2	120.8 (2)	C9—C8—C7	119.7 (3)
N3—C2—N4	121.6 (2)	C9—C8—H8	120.1
N3—C2—C1	121.5 (2)	C7—C8—H8	120.1
N4—C2—C1	116.92 (19)	C8—C9—C10	120.4 (3)
N3—C3—C4	123.6 (2)	C8—C9—H9	119.8
N3—C3—Br1	117.62 (16)	C10—C9—H9	119.8
C4—C3—Br1	118.81 (18)	C5—C10—C9	119.8 (3)
N2—C4—C3	121.2 (2)	C5—C10—H10	120.1
N2—C4—H4A	119.4	C9—C10—H10	120.1
			
C4—N2—C1—N1	178.7 (2)	N3—C3—C4—N2	0.3 (4)
C4—N2—C1—C2	−1.1 (3)	Br1—C3—C4—N2	−178.49 (19)
C3—N3—C2—N4	−177.5 (2)	C2—N4—C5—C10	−59.7 (3)
C3—N3—C2—C1	2.3 (3)	C2—N4—C5—C6	123.5 (3)
C5—N4—C2—N3	−3.5 (4)	C10—C5—C6—C7	1.9 (4)
C5—N4—C2—C1	176.6 (2)	N4—C5—C6—C7	178.8 (2)
N2—C1—C2—N3	−0.9 (3)	C5—C6—C7—C8	0.1 (4)
N1—C1—C2—N3	179.4 (2)	C6—C7—C8—C9	−1.6 (5)
N2—C1—C2—N4	179.0 (2)	C7—C8—C9—C10	0.9 (5)
N1—C1—C2—N4	−0.8 (3)	C6—C5—C10—C9	−2.6 (4)
C2—N3—C3—C4	−2.1 (3)	N4—C5—C10—C9	−179.4 (2)
C2—N3—C3—Br1	176.66 (16)	C8—C9—C10—C5	1.2 (4)
C1—N2—C4—C3	1.3 (4)		

### Hydrogen-bond geometry (Å, °)

**Table:**

*D*—H···*A*	*D*—H	H···*A*	*D*···*A*	*D*—H···*A*
N1—H1B···Cg1^i^	0.86	2.63	3.436 (3)	157
N1—H1A···N2^ii^	0.86	2.22	3.084 (3)	169

Symmetry codes: (i) *x*, −*y*+1/2, *z*−1/2; (ii) −*x*, −*y*+1, −*z*+1.
